# TMEM16A/ANO1 is differentially expressed in HPV-negative versus HPV-positive head and neck squamous cell carcinoma through promoter methylation

**DOI:** 10.1038/srep16657

**Published:** 2015-11-13

**Authors:** Ronak Dixit, Carolyn Kemp, Scott Kulich, Raja Seethala, Simion Chiosea, Shizhang Ling, Patrick K. Ha, Umamaheswar Duvvuri

**Affiliations:** 1 Department of Otolaryngology, University of Pittsburgh, Pittsburgh, Pennsylvania USA; 2 VA Pittsburgh Healthcare System, Pittsburgh, Pennsylvania USA; 3 Department of Pathology, University of Pittsburgh, Pittsburgh, Pennsylvania USA; 4 Department of Otolaryngology, Johns Hopkins University School of Medicine, Baltimore, Maryland USA

## Abstract

Head and neck squamous cell carcinoma (HNSCC) has a variety of causes. Recently, the human papilloma virus (HPV) has been implicated in the rising incidence of oropharyngeal cancer and has led to variety of studies exploring the differences between HPV-positive and HPV-negative HNSCC. The calcium-activated chloride channel TMEM16A is overexpressed in a variety of cancers, including HNSCC, but whether or not it plays different roles in HPV-positive and HPV-negative HNSCC is unknown. Here, we demonstrate that TMEM16A is preferentially overexpressed in HPV-negative HNSCC and that this overexpression of TMEM16A is associated with decreased patient survival. We also show that TMEM16A expression is decreased in HPV-positive HNSCC at the DNA, RNA, and protein levels in patient samples as well as cell lines. We demonstrate that the lower levels of TMEM16A expression in HPV-positive tumors can be attributed to both a combination of copy number alteration and promoter methylation at the DNA level. Additionally, our cellular data show that HPV-negative cell lines are more dependent on TMEM16A for survival than HPV-positive cell lines. Therefore, we suspect that the down-regulation of TMEM16A in HPV-positive HNSCC makes TMEM16A a poor therapeutic target in HPV-positive HNSCC, but a potentially useful target in HPV-negative HNSCC.

Head and neck squamous cell carcinoma (HNSCC) represents the sixth most common cancer in the world and has an overall five-year survival of approximately 50%^1^. Historically, risk factors for this disease include alcohol and tobacco use^2^. More recently, however, HNSCC is occurring in patients without these traditional risk factors. The human papillomavirus (HPV) has been implicated as the causal agent in the rising incidence of HNSCC of the oropharynx^3^.

While HPV-positive and HPV-negative HNSCC are indistinguishable upon initial presentation, there are important distinctions to be made between these two classifications of tumors. For example, HPV-negative HNSCC occurs at various sites in the head and neck, but HPV-positive HNSCC generally arises in the oropharynx^4^. In the United States, the incidence of HPV-negative HNSCC has been decreasing due to decreasing rates of tobacco use, while the incidence of HPV-positive HNSCC has risen dramatically^4^. Importantly, when compared to patients with HPV-negative tumors, patients with HPV-positive HNSCC enjoy improved overall survival and oncologic control^5^.

TMEM16A (also called TAOS2, ANO1, and DOG1), a calcium-activated chloride channel^6^, has been shown to be overexpressed in numerous cancers, including esophageal^7^, gastrointestinal stromal tumors^8^, and HNSCC^9^. The *TMEM16A* gene is located in the chromosomal band 11q13, which is frequently amplified in breast, bladder, esophageal, and head and neck cancers^10^. In HNSCC, TMEM16A has been shown to enhance tumor proliferation via the RAS-RAF-ERK-CCND1 pathway^9^, and a decrease in TMEM16A expression via promoter methylation has been implicated in a shift of HNSCC activity from tumor growth toward metastatic spread^11^. Additionally, overexpression of TMEM16A correlates with decreased patient survival in HNSCC^9^.

While TMEM16A has been found to be a poor prognostic indicator and a possible therapeutic target in HNSCC, no study has compared the role of TMEM16A in HPV-positive and HPV-negative HNSCC. Here, we show that TMEM16A is overexpressed in HPV-negative HNSCC, but not HPV-positive HNSCC. We demonstrate that overexpression of TMEM16A is associated with poor outcome in patients with HPV-negative tumors, but not necessarily in those with HPV-positive cancer. Additionally, we show that TMEM16A expression is in part governed by methylation in both HPV-negative and HPV-positive tumors, although HPV-positive tumors demonstrate higher levels of promoter methylation. Lastly, we demonstrate that cancer cell lines derived from HPV-negative tumors are more reliant on TMEM16A for survival than HPV-positive cell lines. Taken together, these results demonstrate that TMEM16A may prove to be a novel therapeutic target for HPV-negative, but not HPV-positive, HNSCC.

## Results

### TMEM16A is overexpressed in HPV-negative HNSCC

**Figure 1:**
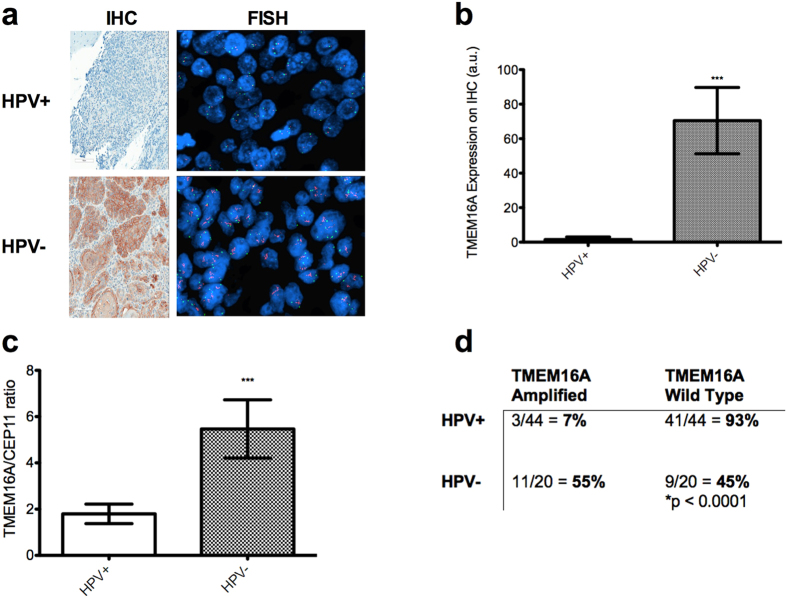
HPV-negative HNSCC expresses more TMEM16A than HPV-positive TMEM16A in primary tumor samples. (**a**) Example IHC (200×, left) and FISH (right) analyses of TMEM16A in head and neck squamous cell carcinoma. The top half of the image represents HPV-positive tumors, with a low degree of IHC and FISH staining. The bottom half, which represents HPV-negative tumors, shows much more staining for TMEM16A in both IHC and FISH. (**b**) TMEM16A protein expression is significantly higher in HPV-negative tumors than HPV-positive patients on IHC (arbitrary units). (**c**) On FISH, TMEM16A/CEP11 ratio is significantly higher in HPV-negative tumors than HPV-positive ones. (**d**) Percent of tumor samples with amplified TMEM16A (defined as TMEM16A/CEP11 ratio > 2.5) and wild type, stratified by HPV-status. HPV-negative tumors were much more likely to have amplification of TMEM16A than HPV-positive ones.

We analyzed tissue samples of patients at our institution using both immunohistochemistry (IHC) and fluorescent *in situ* hybridization (FISH) analysis (Fig. 1a). In our institution’s tissue microarray (TMA), 52 samples stained positively for p16^12^ and were regarded as HPV-positive. 20 tumors were HPV-negative. A semi-quantitative method was used to score TMEM16A protein expression on immunohistochemistry for 44 of the HPV-positive tumors and 20 HPV-negative ones. HPV-negative tumors scored significantly higher on TMEM16A protein expression than HPV-positive tumors (70.46 vs 1.509, p < 0.0001; Fig. 1b).

We then wanted to analyze *TMEM16A* gene amplification by FISH. This was done by taking the ratio of TMEM16A signal intensity (signal-to-nucleus ratio) to CEP11 intensity (centromere-to-nucleus ratio). A total of 44 HPV-positive and 20 HPV-negative tumors had this data available. HPV-negative tumors had a significantly higher TMEM16A/CEP11 ratio than HPV-positive tumors (5.465 vs 1.795, p < 0.001; Fig. 1c). When quantifying the degree to which HPV-negative and HPV-positive tumors had *TMEM16A* gene amplification (defined as TMEM16A/CEP11 ratio > 2.5), chi-squared analysis showed that 55% of HPV-negative tumors showed *TMEM16A* amplification, while only 7% of HPV-positive tumors showed it (p < 0.0001; Fig. 1d).

### TMEM16A overexpression correlates with decreased survival in HPV-negative tumors

**Figure 2:**
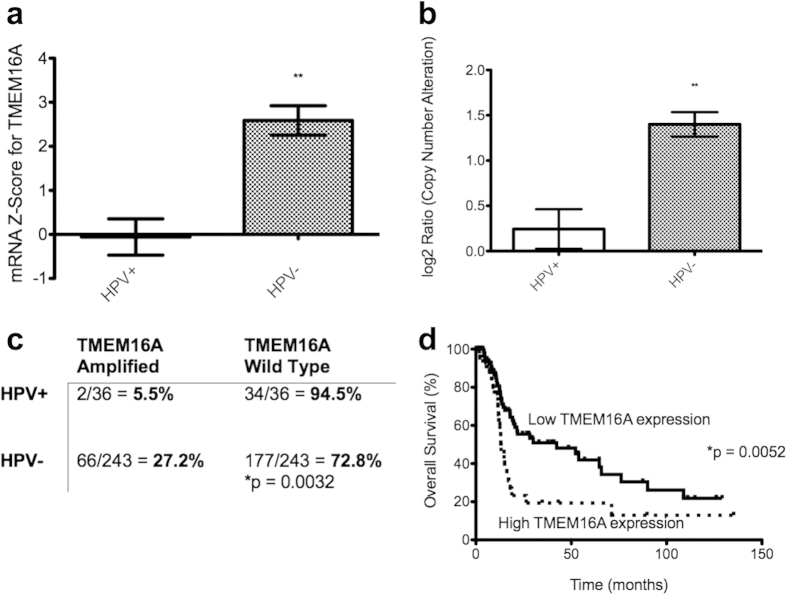
Higher expression of TMEM16A in HPV-negative patients correlates with decreased survival in TCGA. (**a**) Using data from TCGA, mRNA Z-scores for TMEM16A were found to be significantly higher in HPV-negative tumors than HPV-positive ones. (**b**) Similarly, DNA log_2_ ratios for *TMEM16A* gene are significantly higher in HPV-negative tumors than HPV-positive ones. (**c**) The *TMEM16A* gene is much more likely to be amplified (defined as log_2_ ratio > 2) HPV-negative tumors than HPV-positive ones. (**d**) In patients with HPV-negative HNSCC, overexpression of TMEM16A (defined as mRNA Z-score >2) was associated with significantly decreased survival, a pattern that was not seen with HPV-positive HNSCC (see Supplementary Fig. S1).

In order to determine if the results at our institution are generalizable, we carried out similar analysis using data from The Cancer Genome Atlas (TCGA). Stratification of TCGA data revealed 36 HPV-positive tumors and 243 HPV-negative tumors. The mRNA Z-Score is defined as the relative mRNA expression of an individual gene to the gene’s expression distribution in a reference population. In this case, the reference population consists of tumors of the same type (i.e. HNSCC) in TCGA that are known to be diploid for TMEM16A. When comparing mRNA Z-Scores by t-test, HPV-negative tumors were found to express significantly more TMEM16A mRNA than HPV+ tumors (2.588 vs −0.05782, p = 0.003; Fig. 2a).

We next wanted to test for differences in the level of *TMEM16A* amplification. DNA copy number alterations (CNA) in TCGA are calculated using log_2_ ratios of a tumor’s DNA to a reference normal DNA. A log_2_ ratio of 0 indicates no up-regulation (i.e. diploid), and a positive log_2_ ratio is considered gain, with a value of 1 representing a full doubling of copy number. When comparing log_2_ ratios, HPV-negative tumors showed significantly more CNA for TMEM16A than HPV-positive tumors (log_2_ ratio = 1.400 vs 0.2434, p = 0.0016; Fig. 2b). When we quantified the degree to which HPV-negative and HPV-positive tumors showed *TMEM16A* gene amplification (defined as log_2_ ratio > 2), we found that 28.3% of HPV-negative tumors showed *TMEM16A* amplification, while only 5.5% of HPV-positive tumors showed it (p = 0.0019; Fig. 2c).

TCGA also carries survival data for 16 HPV-positive and 143 HPV-negative HNSCC patients. Using a TMEM16A mRNA Z-Score of 2 as the threshold for high TMEM16A expression, survival was significantly decreased in the HPV-negative cohort in those with high TMEM16A expression (median survival = 13.24 months vs 42.32 months, p = 0.0052; Fig. 2d). This survival difference was not significant in the HPV-positive cohort (p = 0.5598, Supplementary Fig. S1); though, it should be noted that only one patient in the HPV-positive cohort had a TMEM16A mRNA Z-Score greater than 2.

### TMEM16A is regulated by promoter methylation

**Figure 3:**
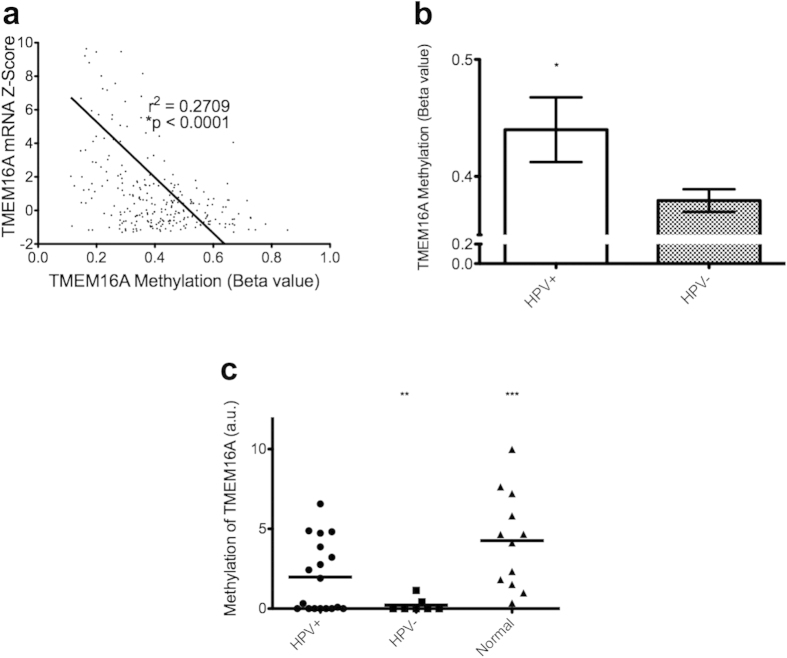
HPV-negative HNSCC shows significantly decreased methylation of *TMEM16A* than HPV-positive HNSCC. (**a**) Using data from TCGA, correlating mRNA expression with methylation shows that degree of methylation correlates with TMEM16A expression with in all data tumors combined. (**b**) In TCGA, level of methylation as measured by HM450 array is significantly lower in HPV-negative tumors. (**c**) qMSP data from our institution’s tissue samples shows that the promoter region of TMEM16A is significantly less methylated in HPV-negative tumors (N = 7) than both HPV-positive tumors (N = 18) and normal pharynx tissues (N = 12).

Since we found that HPV-negative tumors express more TMEM16A than HPV-positive ones, we next aimed to determine if promoter methylation might explain this difference. In TCGA, methylation data was available for all 36 HPV-positive tumors and 242 of the 243 HPV-negative tumors. Methylation level (beta value) of a single CPG island within the promoter region of the *TMEM16A* gene was determined by the Infinium HM450 BeadChip Kit. When analyzing all samples together, methylation negatively correlates with mRNA expression (r^2^ = 0.2709, p < 0.0001; Fig. 3a). Next, we found that the average beta value for HPV-negative tumors was significantly lower than that of HPV-positive tumors (0.3794 vs 0.4399, p = 0.0269; Fig. 3b) indicating less overall methylation in HPV-negative tumors.

We found similar results in our own institution’s tumor samples, in which 18 of the HPV-positive and seven of the HPV-negative tumors could be analyzed by qMSP as described below. These samples were also compared to 12 normal pharynx specimens. Pairwise comparison revealed that the level of methylation in HPV-negative tumors (mean = 0.2243 a.u.) was significantly less than that of HPV-positive tumors (mean = 1.977 a.u., p = 0.0049; Fig. 3c) or normal tissue (mean = 4.260 a.u., p = 0.0008; Fig. 3c).

### HPV-negative tumors are dependent on TMEM16A for survival

**Figure 4:**
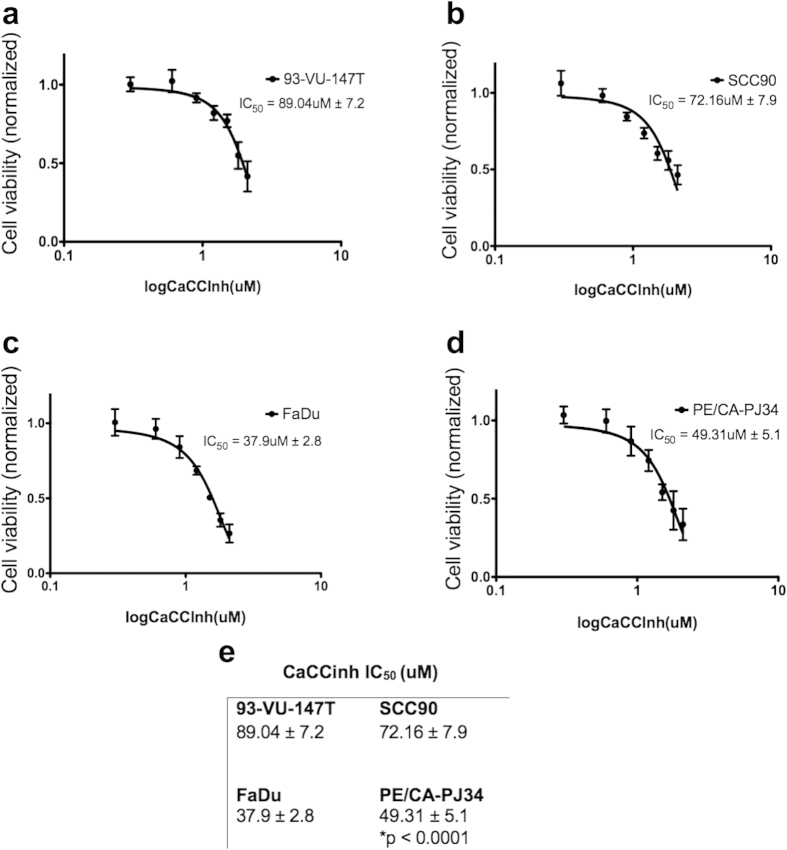
TMEM16A is important for cellular survival in HPV-negative, but not HPV-positive cell lines. (**a**–**d**) Cell survival study with varying concentrations of the small molecule inhibitor of TMEM16A, CaCCinh: The two HPV-positive cell lines, (**a**) 93-VU-147T and (**b**) SCC90, had much higher IC_50_s for CaCCInh than the HPV-negative cell lines, (**c**) FaDu and (**d**) PE/CA-PJ34. (**e**) Table comparing the IC_50_s of each cell line.

To determine the role of TMEM16A in tumor survival, we measured cell viability in response to increasing concentrations of a small molecule inhibitor of TMEM16A, here called CaCCinh^13^, in two HPV-positive and two HPV-negative cell lines. The response to CaCCinh concentrations ranging from 0 uM to 128 uM in HPV-positive SCC-90 and 93-VU-147T cells was compared to that of HPV-negative FaDu and PE/CE-PJ34 cells using a short-term survival assay. The respective half maximal inhibitory concentrations (IC_50_) for 93-VU-147T (Fig. 4a) and SCC90 (Fig. 4b) cells were 89.04 uM and 72.16 uM, while those of FaDu (Fig. 4c) and PE/CA-PJ34 (Fig. 4d) cells were 37.9 uM and 49.31 uM, respectively. Using the Extra sum-of-squares F test, there was a significant difference between the IC_50_s of the different cell lines (p < 0.0001; Fig. 4e). Comparing the IC_50_s of the two HPV-negative cell lines to that of the two HPV-positive cell lines reveals that HPV-negative cells are much more sensitive to CaCCinh (Fig. 4e), and therefore rely on TMEM16A for survival more than HPV-positive cells.

**Figure 5:**
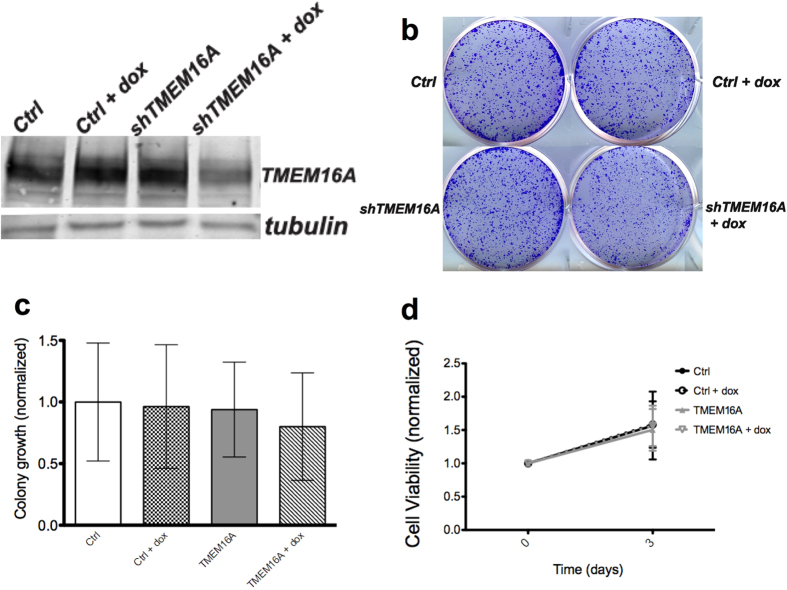
Knockdown of TMEM16A by targeting shRNA does not decrease proliferation or survival in an HPV-positive cell line. (**a**) Western Blot showing targeted knockdown of TMEM16A using doxycycline-induced shRNA in the HPV-positive 93-VU-147T cell line. (**b**) Representative photo of colony formation assay, showing that knockdown of TMEM16A does not decrease colony formation. (**c**) Knockdown of TMEM16A in the HPV-positive 93-VU-147T cell line does not decrease colony formation. (**d**) In 93-VU-147T cells, knockdown of TMEM16A does not result in decreased cell viability compared to control groups in the CellTiter-Glo assay.

Since we found that HPV-positive HNSCC cell lines are more resistant to small molecule inhibition of TMEM16A by CaCCinh, we opted to test this resistance further by knocking down TMEM16A. Using doxycycline-induced non-targeting shRNA (“ctrl”; Fig. 5a) and doxycycline-induced shRNA against TMEM16A (“shTMEM16A”; Fig. 5a), we performed a colony formation assay using the HPV-positive 93-VU-147T cell line to test if knockdown of TMEM16A would result in decreased colony growth. Two weeks after plating colonies on agar, proliferation was assessed by staining with crystal violet (Fig. 5b) and by measuring total area of colonization using ImageJ software. ANOVA found no significant effect in colony growth by group (p > 0.05), and post hoc analysis failed to show any specific difference between groups (p > 0.05; Fig. 5c). Furthermore, we found that knocking down TMEM16A did not result in decreased survival in 93-VU-147T cells in short-term survival assays (Fig. 5d). We used similar methods to knock down TMEM16A in HPV-negative FaDu cells (“TMEM16A KD”; Fig. S2a). The resulting colony formation assay (Fig. S2b) demonstrated significantly less growth in the FaDu cells with TMEM16A knocked down with shRNA (normalized area = 1.00 vs 0.395, p = 0.0358; Fig. S2c). Using siRNA to knock down TMEM16A in HPV-negative PE/CA-PJ34 cells (“TMEM16A KD”; Fig. S2d), we also saw significantly decreased growth in colonies with TMEM16A knocked down (normalized area = 1.00 vs 0.804, p = 0.0002; Figs S2e and S2f).

**Figure 6:**
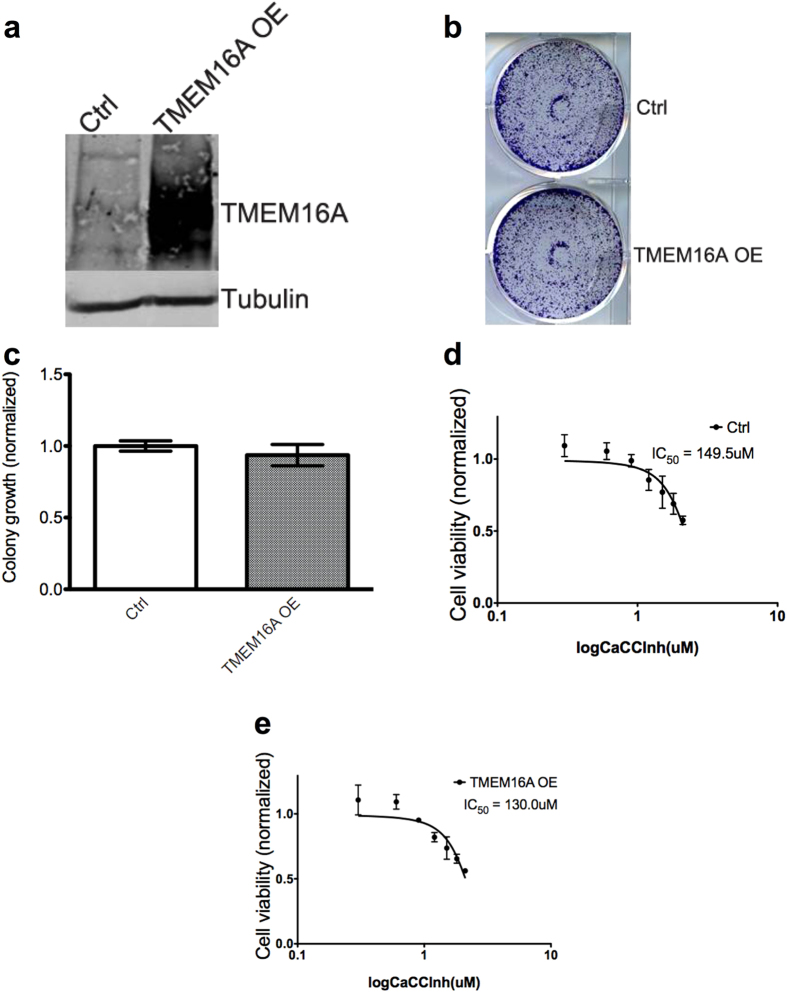
Overexpression of TMEM16A in an HPV-positive cell line confers no increase in proliferation or survival. (**a**) Western Blot showing forced overexpression of TMEM16A in the HPV-positive SCC90 cell line. (**b**) Representative photo of colony formation assay, showing that forced overexpression of TMEM16A does not confer increased colony formation. (**c**) Forced overexpression of TMEM16A in the HPV-positive SCC90 cell line does not confer enhanced proliferation. (**d**,**e**) In SCC90 cells, forced overexpression of TMEM16A does not result in resistance to CaCCinh, as evidenced by the nearly identical IC_50_ to the wild type SCC90 cell line.

After finding that neither inhibition nor knockdown of TMEM16A results in decreased colony formation or cell survival in an HPV-positive cell line, we wanted to test if overexpression of TMEM16A would positively impact survival in an HPV-positive cell line. Using viral transfection, TMEM16A was overexpressed in HPV-positive SCC90 cells (“TMEM16A OE”; Fig. 6a) and a colony formation assay was performed (Fig. 6b). Overexpression of TMEM16A in SCC90 showed an insignificant difference in area of colony formation (normalized area = 1.00 vs 0.9362, p = 0.4826; Fig. 6c). We found that the respective IC_50_s of the wild type SCC90 cells and SCC90 cells with TMEM16A overexpression to CaCCinh were 149.5 uM and 130.0 uM (Fig. 6d,e), an insignificant difference as calculated by the Extra sum-of-squares F test (p = 0.5266). The results of these experiments show that TMEM16A overexpression does not confer enhanced survival to HPV-positive cells.

## Discussion

TMEM16A/ANO1 has been found to be overexpressed in numerous cancers, including esophageal, gastrointestinal stromal tumors, and head and neck cancers^7^,^8^,^9^. Previous studies on this protein in HNSCC have shown that overexpression leads to activation of the RAS-RAF-MEK-ERK1/2 pathway, increased cellular proliferation, and decreased patient survival^9^. Additionally, methylation of its gene promoter region has been shown to decrease expression, and this decreased expression may lead to a shift from cellular proliferation to metastasis^11^. Therefore its importance as a potential therapeutic target in HNSCC has been established.

This is the first study to compare the role that TMEM16A plays in HPV-positive and HPV-negative HNSCC. Our analysis of TCGA as well as our own institution’s tissue microarray (TMA) data clearly indicates that TMEM16A is significantly more highly expressed in HPV-negative HNSCC than in HPV-positive. Previous studies have shown that overexpression of TMEM16A is associated with decreased survival in HNSCC^9^. Our analysis of TCGA shows that this is likely the case only for patients with HPV-negative tumors. In the HPV-positive cohort, TMEM16A overexpression did not lead to a decrease in survival. However, since TCGA has limited survival data available for the HPV-positive cohort and since the HPV-positive cohort as a whole expresses low levels of TMEM16A, only one patient in this group was considered to overexpress TMEM16A. Thus, this survival difference should be re-evaluated once more survival data is available from TCGA or other databases. It is well known that patients with HPV-positive HNSCC have improved overall survival when compared to those with HPV-negative HNSCC^5^. The differential expression of TMEM16A may help to explain this survival difference and is an avenue of study to be explored in the future.

TMEM16A expression has been shown to be regulated by promoter methylation^11^. Since data from TCGA and TMA showed decreased expression in HPV-positive patients, we sought to determine if degree of methylation differs between HPV-positive and HPV-negative tumors. Indeed, we did find that the *TMEM16A* gene has significantly less promoter methylation in HPV-negative tumors in both TCGA and our institution’s TMA. This difference in methylation, in combination with the difference in gene amplification, helps to explain why TMEM16A is more highly expressed in HPV-negative HNSCC.

When working with HPV-negative and HPV-positive cell lines *in vitro*, we saw the importance of TMEM16A for survival in HPV-negative cell lines. We found concurring results when inhibiting TMEM16A either with CaCCinh, a small molecule inhibitor, or by knockdown of TMEM16A with either doxycycline-induced shRNA or siRNA. The HPV-negative cell lines, FaDu and PE/CA-PJ34, were much more sensitive to CaCCinh than the HPV-positive cell lines, SCC90 and 93-VU-147T. Using colony formation assays, we found that knockdown of TMEM16A did not alter colony formation in the HPV-positive 93-VU-147T cells, but did decrease colony formation in HPV-negative FaDu and PE/CA-PJ34 cells. Similarly, overexpression of TMEM16A in HPV-positive SCC90 cells did not confer a survival benefit, showing that this is not a protein that promotes survival in HPV-positive tumors.

In conclusion, we have shown that TMEM16A plays a more important role in HPV-negative HNSCC than in HPV-positive HNSCC. We have determined that 1) HPV-negative tumors express more TMEM16A at the DNA, mRNA, and protein level; 2) only patients with HPV-negative HNSCC have decreased survival when TMEM16A is overexpressed; 3) HPV-negative tumors have decreased promoter methylation of *TMEM16A* compared to HPV-positive ones; 4) HPV-negative cell lines are more sensitive to pharmacologic inhibition of TMEM16A than HPV-positive cell lines; 5) Knockdown of TMEM16A decreases colony formation in HPV-negative cell lines but not HPV-positive cell lines; and 6) TMEM16A overexpression does not confer an increase in colony formation in HPV-positive cell lines. Taken together, these results suggest that TMEM16A may be a viable target for therapy in HPV-negative tumors, but not necessarily for head and neck squamous cell carcinoma that has been caused by HPV.

## Materials and Methods

### Primary Tissue Samples

All experiments were carried out after obtaining approval from the University of Pittsburgh Institutional Review Board, and all experiments were performed in accordance with guidelines set out by that institution. Before obtaining primary tissue samples, informed consent was obtained from each subject. As described by Shiwarski *et al.*^11^, primary pharynx tissues were collected for tissue microarray (TMA). Tissue samples were formalin fixed and paraffin embedded from patients who underwent curative surgery for HNSCC. Immunohistochemistry for p16 (G175–405; BD Pharmingen, San Diego, CA), as a surrogate marker for HPV, was performed as per manufacture’s protocol. Cases were considered positive if more than 70% of tumor cells showed diffuse strong cytoplasmic and nuclear staining. Staining was also performed with anti-TMEM16A antisera (clone SP31 ThermoFisher). Slides were scored using a semi-quantitative system.

Fluorescence *in situ* hybridization (FISH) studies were carried out on the TMA samples using a probe for the centromere of chromosome 11 (CEP11) labeled with SpectrumGreen (Abbott Molecular) and a probe prepared from a BAC clone (RP11-805J14; CHRI) and labeled by nick translation with SpectrumOrange^9^. A minimum of 30 cells per case were analyzed, and a quantitative system comparing signal-to-nucleus ratio and centromere-to-nucleus ratio was used to describe the degree of gene amplification.

### The Cancer Genome Atlas

Using the Head and Neck Squamous Cell Carcinoma in Revision database from The Cancer Genome Atlas (TCGA) (http://cancergenome.nih.gov/), parameters of 279 HNSCC patient samples and associated clinical data were analyzed. The data were accessed using the cBioPortal^14^,^15^ for Cancer Genomics, via R statistics software^16^. Data collected include patient HPV status, patient survival, relative levels of TMEM16A mRNA expression assessed by RNA Seq Version 2, DNA copy number alterations assessed via array-based Comparative Genomic Hybridization (aCGH), and DNA methylation profiling using Illumina Infinium HumanMethylation450 BeadArray.

### Cell Culture

All cell lines were used after genotype verification. HPV-positive cells included 93-VU-147T^17^ (gift from Dr. Hans Joenje, VU Medical Center Van der Boechorststraat 7, The Netherlands) and UPCI:SCC90^18^. HPV-negative PE/CA-PJ34 and FaDu cells^19^ were obtained from Sigma Aldrich and American Type Culture Collection, respectively. All cell lines were grown in DMEM with 10% Fetal Bovine serum.

### Stable cell line generation

Stable cell lines either overexpressing or knocking down TMEM16A were generated using retroviral transduction as described previously^11^. Briefly, retroviral particles were created by transfecting Plat-A cells (ATCC) with the appropriate plasmids. Viral supernatant was then used to transduce the requisite cell lines. Antibiotic selection with puromycin (1 ug/ml) was used to select for transduced cells. Stable cells were used for 10 passages, and then discarded. Similar methods were used to achieve knockdown of TMEM16A in a doxycycline-induced shRNA system as well as for siRNA knockdown.

### Cell Viability Assay

For proliferation and viability analysis, cells were plated in black walled 96-well optical plates at 5 × 10^3^ cells/well. Cells were treated with various concentrations of CaCCinh-A01^13^ (referred to as “CaCCinh” throughout this study), a small molecule inhibitor of TMEM16A. 48 hours after treatment, the CellTiter-Glo Assay (Promega) was used according to the manufacturer’s directions to establish proliferation viability for each cell line. Each experiment was run in triplicate unless otherwise stated.

### Colony formation assay

As described previously^11^, 5 × 10^4^ cells suspended in 0.7% agar solution were plated in a 35-mm dish on top of 1.4% agar. Colonies were stained with crystal violet after 2 weeks of growth, and then area was measured after 3 weeks. Total area of colony formation was calculated using ImageJ software. Normalized areas are reported here.

### Bisulfite treatment and Quantitative methylation-specific PCR

The method of methylation analysis was similar to that described by Shiwarski *et al.*^11^. Briefly, the EpiTect Bisulfite Kit (Qiagen) was used to convert unmethylated cytosines in DNA to uracil according to the manufacturer’s instructions. Quantitative methylation-specific PCR (qMSP) was carried out in a 7900 sequence detector (Perkin-Elmer Applied Biosystems, Carlsbad, CA) and analyzed by a sequence detector system (SDS 2.3; Applied Biosystems). The *TMEM16A* qMSP primer sequences designed were: Forward 5′- AGGATCGTAGCGTTTATATTA -3′, and Reverse 5′- CGCGACCCTCCCGCC -3′. The *TMEM16A* qMSP probe sequence was 6FAM 5′- CGCACTCACCGTACCCTCG -3′ TAMRA.

Leukocyte DNA from a healthy individual was methylated *in vitro* with excess *SssI* methyltransferase (New England Biolabs, Inc., Ipswich, MA) to generate completely methylated DNA. Serial dilutions (30–0.003 ng) of this bisulfite-treated methylated DNA were used to construct a calibration curve. All data points were within the range of sensitivity and reproducibility of the assay based on the calibration curve. The methylation levels in each sample were determined as a ratio of qMSP-amplified gene to β-actin (reference gene) and then multiplied by 1000 for easier tabulation (average value of gene triplicates divided by the average value of β-actin triplicates × 1000).

### Statistical Analysis

Statistical analysis was performed using GraphPad Prism 5. All data are reported as mean ± SEM unless stated otherwise. For continuous variables, t-tests and ANOVA were performed where appropriate. For categorical data, chi-squared test was used to determine signifance. Correlations were calculated using Pearson’s r^2^. Survival analysis was performed using the Kaplan-Meier method and log-rank testing. For cell viability assays, IC_50_ was calculated by constraining maximal response to 100% and minimal response to 0%. For colony formation assays, all areas are reported with the control group normalized to 1.

## Additional Information

**How to cite this article**: Dixit, R. *et al.* TMEM16A/ANO1 is differentially expressed in HPV-negative versus HPV-positive head and neck squamous cell carcinoma through promoter methylation. *Sci. Rep.* **5**, 16657; doi: 10.1038/srep16657 (2015).

## Supplementary Material

Supplementary Figures S1 and S2
