# Supplementary material for: TMEM16A/ANO1 is differentially expressed in HPV-negative versus HPV-positive head and neck squamous cell carcinoma through promoter methylation
Source: Sci Rep. 2015 Nov 13;5:16657. doi: 10.1038/srep16657 (PMC4643216; doi:10.1038/srep16657)
Supplement: Supplementary Figures S1 and S2 [file srep16657-s1.pdf]

TMEM16A/ANO1 is differentially expressed in HPV-negative versus HPV-positive head and neck squamous cell carcinoma through promoter methylation.

Ronak Dixit MD, Carolyn Kemp BS, Scott Kulich MD, PhD, Raja Seethala MD, Simion Chiosea MD, Shizhang Ling PhD, Patrick K Ha MD, and Umamaheswar Duvvuri MD, PhD

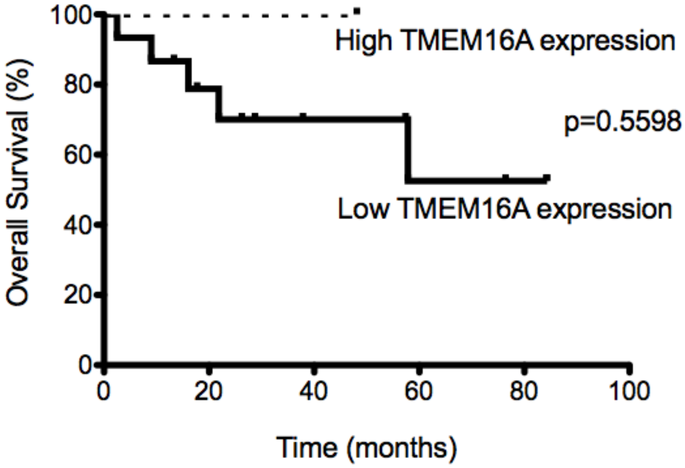


Supplementary Figure S1: Overexpression of TMEM16A is not associated with decreased survival in HPV-positive HNSCC

Unlike the HPV-negative patients in TCGA, the limited available data of patients with HPV-positive tumors shows that the difference in survival between patients who overexpress TMEM16A and those who do not was not statistically significant.  It should be noted that only one patient had a TMEM16A mRNA Z-Score above 2.

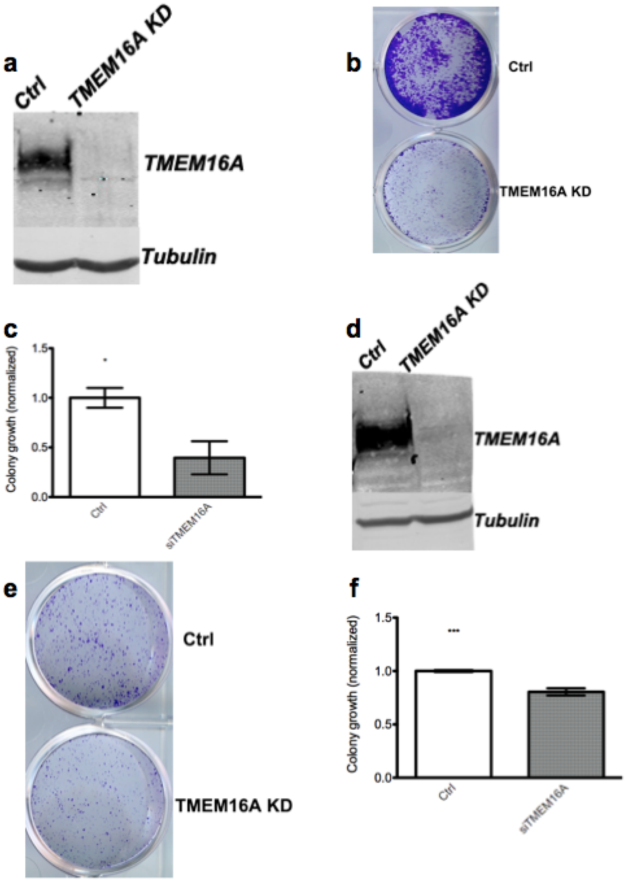


Supplementary Figure S2: Knock down of TMEM16A leads to decreased colony formation in HPV-negative HNSCC cell lines

(a) Western Blot showing knock down of TMEM16A in the HPV-negative FaDu cell line. (b) Representative photo of FaDu colony formation assay, showing that knock down of TMEM16A leads to decreased colony formation. (c) Knockdown of TMEM16A by shRNA significantly reduces colony formation in HPV-negative FaDu cells. (a) Western Blot showing knock down of TMEM16A in the HPV-negative PE/CA-PJ34 cell line. (b) Representative photo of PE/CA-PJ34 colony formation assay, showing that knock down of TMEM16A leads to decreased colony formation. (c) Knockdown of TMEM16A by siRNA significantly reduces colony formation in HPV-negative FaDu cells.
